# Cost-Effective Design of a Miniaturized Zoom Lens for a Capsule Endoscope

**DOI:** 10.3390/mi13111814

**Published:** 2022-10-24

**Authors:** Wen-Shing Sun, Chuen-Lin Tien, Ping-Yi Chen

**Affiliations:** 1 Department of Optics and Photonics, National Central University, Chungli 32001, Taiwan; 2 Department of Electrical Engineering, Feng Chia University, Taichung 40724, Taiwan

**Keywords:** zoom lens, optimization design, capsule endoscopy, relative illuminance

## Abstract

This paper presents a miniaturized design of a 2× zoom lens for application to a one-megapixel image sensor in a capsule endoscope. The zoom lens is composed of four lenses, including three plastic aspheric lenses and one glass spherical lens, and adopts a three-lens group design. This capsule endoscope is mainly for observation of the small intestine, which has a radius of about 12.5 mm. The height of the object is thus set to 12.5 mm. The object surface is designed to be curved surface with a radius of curvature of 15 mm. The focal length of the zoom lens ranges from 1.064 mm to 2.039 mm, and the full angle of view ranges from 60° to 143°, the f-number is F/2.8–F/3.5, the zoom lens is 11.6 mm in length, and the maximum effective diameter of the zoom lens is 6 mm. The zoom lens design is divided into six segments, corresponding to the different magnifications from Zoom 1 to Zoom 6. The magnification ratios are −0.0845, −0.0984, −0.1150, −0.1317, −0.1482, and −0.1690, respectively. Comparing the positions from Zoom 1 to Zoom 6, the maximum optical distortion is −14.89% for the Zoom 1 and 1.45% for the Zoom 6. The maximum vertical video distortion is 8.19% for Zoom 1 and 1.00% for Zoom 6. At a 1.0 field of view, the minimum relative illuminance is 71.8% at a magnification of M = −0.1317. Finally, we perform the tolerance analysis and lens resolution analysis at different zooming positions. Our design can obtain high-quality images for capsule endoscope.

## 1. Introduction

Capsule endoscopy is an important diagnosis system, mainly used to monitor and make recordings of the digestive tract and provide real-time image. There is a tiny wireless camera inside the capsule, which can record images as the capsule passes through the digestive system. Capsule endoscopy was first developed by Dr. Gavriel Iddan in 1999, leading the first generation of capsule endoscopy (M2A) [1]. It was approved by the US Food and Drug Administration in August 2001 and has been certified and approved for listing by the Bureau. The Given (Gastrointestinal Video Endoscope) imaging company was formed to conduct continuous research and development. Since then, other countries have invested in this research, including Japan’s RF system Lab [2] and Olympus [3], South Korea’s Intelligent Micro-system [4], and China’s Jinshan [5] and Ankon Optoelectronics [6].

In 2007, Ou-Yang et al. [7] improved the design of the capsule endoscope. Their design has only two lens groups; the second surface is aspherical, the F-number (F/#) is 2.8, and the aperture size of the first surface is 1.73 mm. The total length of the lens is 3.47 mm, and the full angle of view is 86.26°. In order to address the problem of optical distortion caused by large viewing angles, they added a mirror group glued to the protective optical cover to form a three-piece design with a rear mirror group. The used five aspherical surfaces are used, with the object surface set to be a curved surface in the design. The F-number of that device is 2.8, the aperture size of the first surface is 9.2 mm, the total length of the lens is 10.7 mm, and the full angle of view is 86.26° [8]. In 2011, Jeng et al. [9] designed an annular scanning capsule endoscope in which the first piece is a conical mirror with a radius of 3.35 mm, the last two pieces are aspherical lenses, the F/# is 4.2, the total lens length is 8.855 mm, and the full viewing angle is 60.58°. In 2015, Sheu and Pan et al. [10,11] designed a lens system for a dual-view capsule endoscope. The mirror surface was comprised nine spherical and three aspherical surfaces, for an F/# of 3.5, and a first surface aperture of 5.774 mm. This device has a full front viewing angle of 90° and a rear viewing angle of 260°–290°. The total length of the lens is 11.226 mm. In 2016, Zhang et al. [12] presented an autofocus capsule endoscope system based on a liquid lens. This system can be electronically controlled to autofocus, and without any moving optical elements. In 2020, Chang et al. [13] designed a compact wide-angle (160 deg.) capsule endoscope lens with F-number 2.8 and total track length of 5 mm. The proposed system consists of five elements, all of which employ aspheric surfaces to control optical aberrations. In 2021, Kim et al. [14] reported a plastic endoscopic lens design, providing both f-number of 2.2 and field-of-view of 140°. The modulation transfer function (MTF) is over 20% at 180 lp/mm, and relative illumination is more than 60% in the full field. For the commercial product development, Given Imaging in Israel [15] was the first company to develop diagnostic products for capsule endoscopy. Different products for capsule endoscopy with different specifications were developed for observation of the small intestine (PillCam SB3), esophagus (PillCam ES03) and colon (PillCam COLON 2). However, the lens design for the capsule endoscope is mainly based on a wide-angle lens. There is a lack of research on the zoom lens of capsule endoscope.

As can be seen from the above literature, most capsule endoscope lenses use a fixed focal length due to limited space. There are few articles on the zoom lens design for capsule endoscopes. This paper presents a new capsule endoscope zoom lens design using three plastic aspheric lenses and one glass spherical lens. In addition to increasing the viewing angle, the 2× zoom lens design can also be achieved. Due to the volume restrictions of the endoscope capsule, the total length of the lens must be less than 12 mm. In this work, a 1-megapixel image sensor with a pixel size of 1.4 µm was selected for the zoom lens design that will be applied to capsule endoscopes. Based on a cost-effective optimization design, we expect the proposed design to obtain high-quality images for capsule endoscope.

## 2. Design Methods

The plastic materials are widely used in aspheric lens design of an optical system. Plastic lenses can be produced by injection molding techniques, especially for aspheric surfaces to control aberrations and improve the image quality. Plastic lens has the advantages of low price, lightweight and easy manufacture. The main starting point for any zoom lens design is the selection of specifications. The initial specifications are chosen for a similar patented design or from the optical related literature as the initial design and then they are optimized through the application of optical software. The next step is to evaluate whether the optimized results can meet the design specifications. If they do not meet the requirements, it is necessary to modify the design or change the optimization method. If the target value is not reached, it is necessary to review the specification settings for problems or to change the initial design before optimization. If the design specifications are met, a final tolerance analysis must be carried out to evaluate whether the designed optical system can be mass produced and manufactured.

### 2.1. Design Specifications

In this study, a one-megapixel CMOS image sensor (product model OV9724, 1/9 inch in size) produced by OmniVision was selected. The effective area of the sensor is 1.840 mm × 1.040 mm, and the diagonal length can be calculated to be 2.113 mm, the pixel size is 1.4 µm × 1.4 µm, and the number of effective pixels is 1280 (H) × 720 (V). In our lens design, the main observation target is the small intestine which has an average radius of about 12.5 mm, so this value is set as the object height. Since the purpose of capsule endoscopy is mainly to observe any abnormalities along the intestinal wall, the object surface is not an ideal plane but is relatively close to a curved surface. Therefore, in this study, the object surface is designed to be curved, to more closely conform to the real situation. The radius of curvature of the object surface is assumed to be 15 mm. According to the sensor specifications, its diagonal length is 2.113 mm, and the imaging height can be defined as 1.056 mm. If the height of the object is −12.5 mm, when the zoom 1 magnification is set at the wide-angle end, the zoom lens magnification can be calculated as −0.0845.

The proposed zoom lens design is divided into six segments corresponding to the different magnifications (as expressed in M). The magnification ratios of Zoom 1 at the wide-angle end to Zoom 6 at the telephoto end are −0.0845, −0.0984, −0.1150, −0.1317, −0.1482, and −0.1690, respectively. The zoom lens has two levels of magnification. The short focal length is 1.064 mm at the wide-angle end, and the long focal length is 2.039 mm at the telephoto end. The field of view at the wide-angle end is 143 degrees, and the angle of view at the telephoto end is 60 degrees. The F-number ranges from 2.8 to 3.5. The length of the zoom lens system (from the first side of the lens to the imaging surface) should be less than 11.6 mm, the aperture diameter of the zoom lens should be less than 6 mm, and the principal ray angle on the image side should be less than 14°. Then the selected light source wavelengths are 642.73 nm, 590.86 nm, 542.02 nm, 500.48 nm, and 465.61 nm, respectively. The corresponding weighting factors are 7, 36, 42, 13, and 2, respectively.

When the influence of lens aberration and assembly tolerance is considered, the imaging quality will be reduced. Therefore, a dynamic modulation transfer function (DMTF) of 0.7 is set as the designed spatial frequency during the process of zoom lens optimization. The designed spatial frequency will vary with different F-number values. The F-numbers corresponding to spatial frequencies of 147, 132, 121 lp/mm are 2.8, 3.2, 3.5, respectively, for the different zoom positions. In addition, in order to avoid a large difference between the tangential (Tangential) and the radial (Sagittal) MTF, so the astigmatism is too large, the absolute value of the difference should be less than 10%. The size of the lateral color should be less than one pixel, which is 1.4 µm. The absolute value of the optical distortion is less than 15% at the wide-angle end, and less than 2% at the telephoto end. The TV distortion is less than 10% at the wide-angle end, and less than 1% at the telephoto end. Finally, the relative illuminance required should be greater than 65%. In the lens manufacturing specifications, the thickness of the glass at the center should be greater than 0.8 mm, the edge thickness must be greater than 1.2 mm, and the centering coefficient of the glass lens greater than 0.12. The central thickness of a plastic lens must be greater than 0.8 mm, the edge thickness must be greater than 1.2 mm, and the ratio of the thickest part to the thinnest part of the plastic lens is required to be less than 3.0. The Fujifilm’s patented fixed focus lens [16] is adopted here for the initial design.

### 2.2. Optimization Process

**Figure 1:**
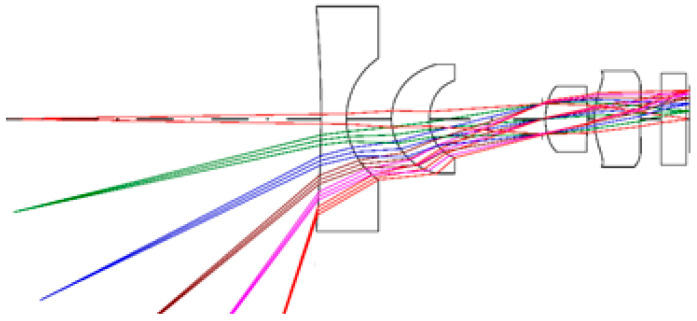
Schematic diagram of a 2× zoom lens design with a glass spherical lens and three plastic aspheric lenses in sequence.

The optical simulation software CODE V is used to optimize the zoom design. First, we set the F/#, light source wavelength, paraxial image height, and then input the initial value. The total length of the lens system, the aperture size of the first surface and the magnification are used as constraints. The variables for the curvature radius, thickness, air spacing, and the refractive index and dispersion coefficient of the material are set. Then, the lens thickness, inter-lens spacing, and glass material selection of general constraints are set, and five (nd, Vd) values from the Schott glass (nd, Vd) diagram are selected as the optimization range. Five plastic materials (nd, Vd) are also selected as the optimization range. After the initial specifications and system size requirements are met, it is necessary to determine the imaging quality. In order to eliminate aberrations and improve the image quality, a miniaturized zoom lens design combined with three plastic aspherical lenses is proposed, as shown in Figure 1. The first group consists of two lenses, the front lens is a glass spherical lens, and the rear lens is a plastic aspherical lens. The second group is comprised of a plastic aspherical lens, and the third group is also a plastic aspherical lens. Finally, an analysis is carried out to find whether the imaging quality of the optimized zoom lens meets the design requirements. If it does meet the imaging quality requirements, the design is completed. If the imaging quality requirements are not met, we go back to the settings of specific constraints and make adjustments to re-execute the optimization process.

## 3. Design Results

### 3.1. Zoom Lens Design Results

**Figure 2:**
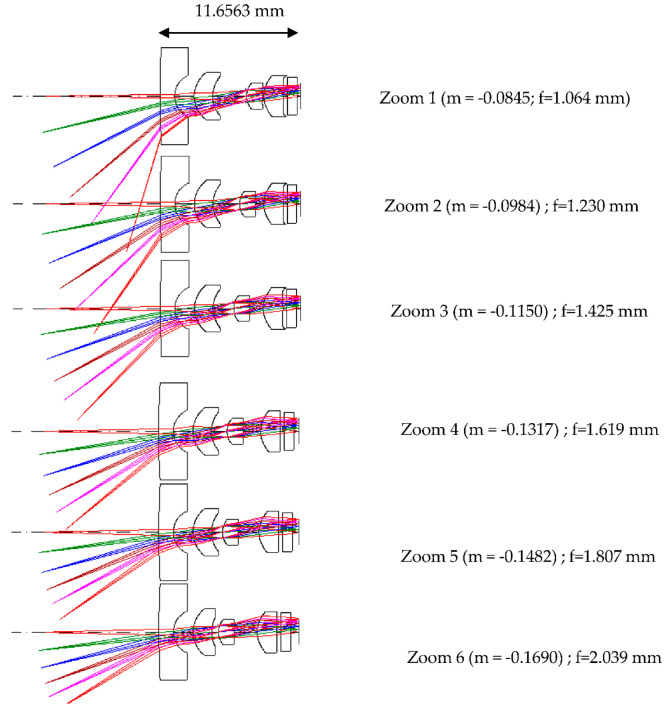
Lens diagrams of the 2× zoom lens at different magnifications.

**Figure 3:**
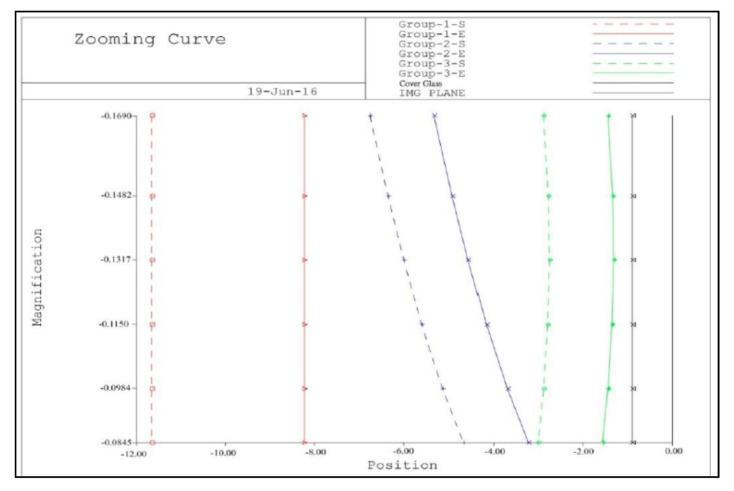
Trajectory diagram of a 2× zoom lens.

Figure 2 shows the lens diagrams of the 2× zoom lens at different magnifications. The main imaging lens group consists of four pieces, one piece of glass material, three pieces of plastic, and finally a piece of protective glass for the sensor. The length of the zoom lens is 11.65 mm. The first piece of glass is a spherical lens with a diameter of 6 mm, and the second, third and fourth plastic pieces are aspheric lenses. Figure 3 shows a trajectory diagram for the three-group zoom lens. The abscissa gives the lens position, the position of 0 represents the imaging surface, and the ordinate shows the magnification. There are three lens groups. The first group is comprised of the first and second lenses, the second group is comprised of the third lens, and the third group is comprised of the fourth lens. When zooming, the first group and the image plane are fixed, and both the second group and the third group move simultaneously. The dotted line indicates the first curve of each group and the solid line the last curve of each group. The air space between each lens group is greater than 0.1 mm. The final design results after optimization of the zoom lens are shown in Table 1.

### 3.2. Analysis of the Main Ray Angle of the Image Square

The paraxial imaging height is designed to be half the diagonal length of the sensor, with a value of 1.056 mm. During the optimization process, six different paraxial imaging heights (0, 0.2464 mm, 0.4797 mm, 0.6888 mm, 0.8671 mm, 1.056 mm) are optimized to finally obtain the angular relationship of image-side chief rays at different paraxial imaging heights with different magnifications during the zooming process.

### 3.3. Image Quality

**Figure 4:**
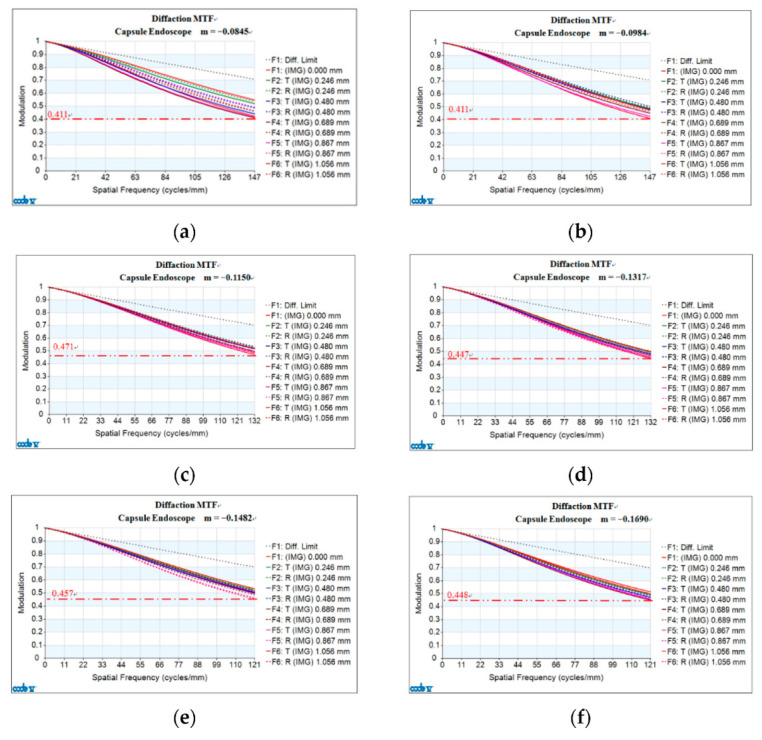
MTF curves for a 2× zoom lens at different magnifications: (**a**) M = −0.0845; (**b**) M = −0.0984; (**c**) M = −0.1150; (**d**) M = −0.1317; (**e**) M = −0.1482; (f) M = −0.1690.

Analysis of the imaging quality of the 2× zoom lens includes the MTF, optical distortion, lateral color, and relative illumination. Figure 4a shows the MTF curve when the magnification of the internal lens is M = −0.0845. The abscissa of the graph gives the spatial frequency, which has a maximum value of 147 lp/mm; the ordinate shows the modulation; the Diff. Limit is the DMTF curve. Six paraxial imaging heights (namely 0.246 mm, 0.480 mm, 0.689 mm, 0.867 mm, 1.056 mm), and tangential (T) MTF and radial (R) MTF were taken at each paraxial imaging height. For the center of the image (h = 0 mm), the tangential MTF is the same as the radial MTF, and there are 12 curves in total. The lowest MTF occurs at the image height of 0.689 mm, and the tangential MTF is 0.411at a spatial frequency of 147 lp/mm. For a magnification of M = −0.0984, the lowest MTF position is located at the image height of 1.056 mm, and the tangential MTF is 0.411 at a spatial frequency of 147 lp/mm, as indicated in Figure 4b. When the magnification of M = −0.1150, the lowest MTF position is located at the image height of 1.056 mm, and the tangential MTF is 0.471 at a spatial frequency of 132 lp/mm, as shown in Figure 4c. For a magnification of M = −0.1317, the lowest MTF is located at the image height of 1.0560 mm, and the tangential MTF is 0.447 at a spatial frequency of 132 lp/mm, as shown in Figure 4d. For a magnification of M = −0.1482, the lowest MTF position is located at an image height of 1.056 mm, the radial MTF is 0.457 at the spatial frequency of 121 lp/mm, as shown in Figure 4e. For a magnification of M = −0.1690, the lowest MTF occurs at the image height of 0.689 mm, the tangential MTF is 0.448 for a spatial frequency of 121 lp/mm, as shown in Figure 4f.

From Zoom 1 to Zoom 2, the F/# is 2.8, the maximum spatial frequency is 147 lp/mm, the minimum modulation transfer function (MTF) is 0.408, and the maximum difference between the absolute values of the tangential MTF and the radial MTF is 0.078. From Zoom 3 to Zoom 4, the F/# is 3.2, the maximum spatial frequency is 132 lp/mm, the minimum MTF is 0.447, and the maximum difference between the absolute values of the tangential MTF and the radial MTF is 0.024. From Zoom 5 to Zoom 6, the F/# is 3.5, the maximum spatial frequency is 121 lp/mm, the minimum MTF is 0.448, and the maximum difference between the absolute values of the tangential MTF and the radial MTF is 0.053. The results also reveal that the maximum optical distortion of the zoom lens varies with different magnifications. Table 2 shows the maximum optical distortion of the zoom lens at different magnifications.

**Figure 5:**
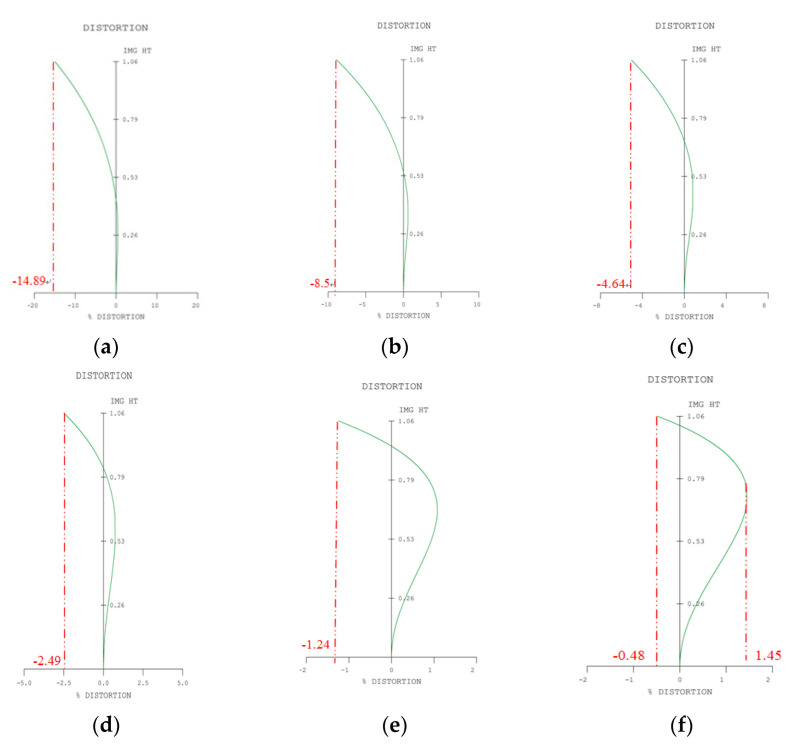
Optical distortion curves of a 2× zoom lens at different magnifications: (**a**) M = −0.0845; (**b**) M = −0.0984; (**c**) M = −0.1150; (**d**) M = −0.1317; (**e**) M = −0.1482; (**f**) M = −0.1690.

Figure 5 shows optical distortion curves of a 2× zoom lens at different magnifications. The horizontal axis gives the optical distortion (%), and the vertical axis shows the paraxial imaging height (mm). A comparison of the horizontal video distortion and vertical video distortion at different magnification positions appears in Table 3. The maximum video distortion for Zoom 1 at the wide-angle end is 8.19%, the maximum video distortion for Zoom 2 is 4.98%, the maximum video distortion for Zoom 3 is 3.08%, and the maximum video distortion for Zoom 4 is 2.07%, The maximum video distortion for Zoom 5 is 1.53%, and the maximum video distortion for Zoom 6 is 1.00%.

**Figure 6:**
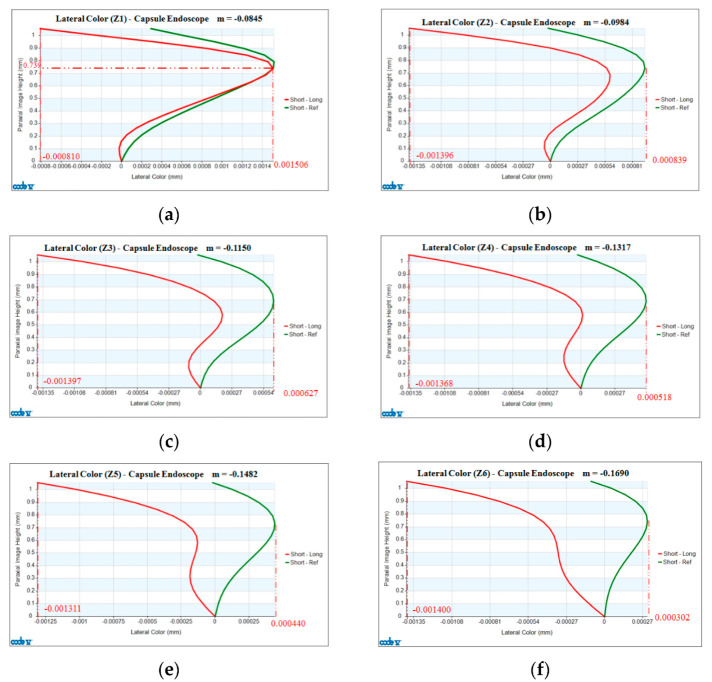
Lateral color curves of a 2× zoom lens at different magnifications: (**a**) M = −0.0845; (**b**) M = −0.0984; (**c**) M = −0.1150; (**d**) M= −0.1317; (**e**) M = −0.1482; (**f**) M= −0.1690.

The design results show the lateral chromatic aberration of a 2× zoom lens is varied with different magnifications. Figure 6a shows the lateral color curve of the zoom lens at a magnification of M = −0.0845. The horizontal axis gives the lateral color (mm), and the vertical axis shows the paraxial imaging height (mm). The red curve in the figure indicates the short wavelength (465.61 nm) and the green curve represents change of lateral color of the long wavelength (642.73 nm) at different paraxial image heights. The maximum absolute value of the lateral color is 0.001506 mm. The green curve indicates the change in the lateral color between the short wavelength and the reference wavelength (542.02 nm) at different paraxial image heights. The maximum absolute value of the lateral color is 0.001506 mm for the red curve and −0.00081 mm for the green curve. Figure 6b shows a graph of the lateral color of the zoom lens at a magnification of M = −0.0984, where the maximum absolute value of the lateral color is −0.001396 mm for the red curve and 0.000839 mm for the green curve. The lateral chromatic aberrations from Figure 6c–f are similar, but the values are different. The comparison of lateral chromatic aberrations of the zoom lens at different magnifications is shown in Table 4. 

**Figure 7:**
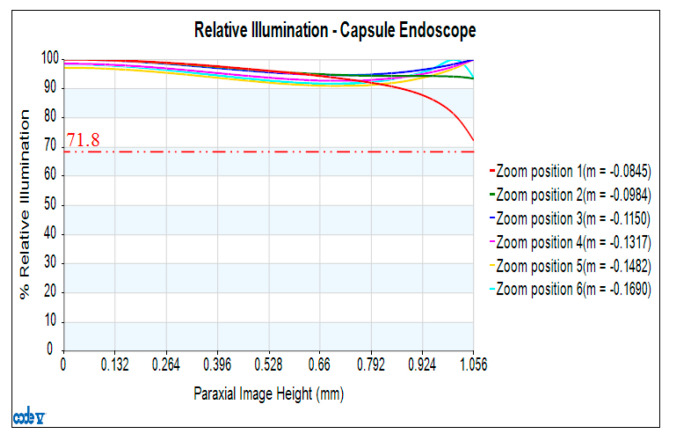
Relative illuminance diagram of a 2× zoom lens.

Figure 7 shows the relative illuminance curves of the zoom lens at magnifications of M = −0.0845, −0.0984, −0.1150, −0.1317, −0.1482, −0.1690, where the horizontal axis gives the paraxial imaging height (mm), and the vertical axis shows the relative illuminance (%). Under the 1.0 field of view and the magnification of M = −0.1317, the minimum value of relative illumination is 71.8%.

## 4. Tolerance Analysis

In addition to making a zoom lens design that meets the specifications, we also need to consider whether the design can be made. Therefore, through tolerance analysis, it is discussed whether any decline in image quality caused by lens manufacturing and assembly errors would be within an acceptable range. The higher the tolerance, the easier the lens is to make. The production equipment of each factory is different, so the tolerance range of each company is different, and it must be determined whether production can be carried out within this range. If the yield is too low, the production cost will increase.

### 4.1. Tolerance Analysis Process

Each tolerance must be specified in a suitable range before the product can be manufactured. The tolerance range used in this study is from Ref. [17]. Since the designed value is set at 40%, the probability of successful manufacture is 2σ (97.7%), to ensure that the MTF performance of the zoom lens at different magnifications and all field angles can be kept above 30%. Therefore, our design method is to reduce the MTF value by 10%. A set of tolerance range is analyzed and compared with the acceptable tolerance range.

### 4.2. Tolerance Analysis Results

**Figure 8:**
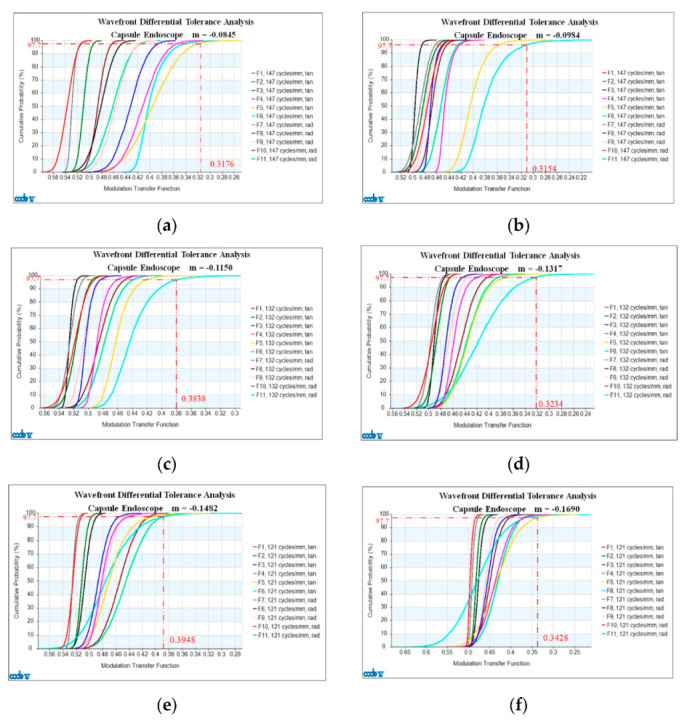
Cumulative probability vs. MTF: (**a**) M = −0.0845; (**b**) M = −0.0984; (**c**) M = −0.1150; (**d**) M = −0.1317; (**e**) M = −0.1482; (**f**) M = −0.1690.

Figure 8a shows a graph of cumulative probability versus MTF for the zoom lens at a magnification of M = −0.0845. The MTF appears on the horizontal axis and the cumulative probability (%) on the vertical axis. Note that of the 11 different color curves (F1 to F11) displayed in the figure, F1, F2, F3, F5 and F6 represent the tangents of the different cumulative probabilities plotted at six different paraxial imaging heights (0, 0.2464 mm, 0.4797 mm, 0.6888 mm, 0.8671 mm, 1.0560 mm) relative to the design and MTF (147 lp/mm). In this figure, F7, F8, F9, F10, and F11 represent the different cumulative probabilities at five different paraxial imaging heights (namely 0.2464 mm, 0.4797 mm, 0.6888 mm, 0.8671 mm, 1.0560 mm, respectively), relative to the design and the radial MTF tolerance (42 lp/mm). The tangential and radial MTF values obtained with this design and the tolerance for a paraxial imaging height of zero are the same. The position selected for the cumulative probability evaluation in the general tolerance analysis is 2σ (97.7%). The tolerance analysis results of 2× zoom lens with magnification of M = −0.0845 at six different paraxial imaging heights (i.e., 0, 0.2464 mm, 0.4797 mm, 0.6888 mm, 0.8671 mm, 1.0560 mm) are obtained. The minimum value of the tangential and radial MTF is 0.3176 with the tolerance. Figure 8b shows values of the cumulative probability versus MTF curve of the zoom lens at a magnification of M = −0.0984. The minimum designed MTF tolerance is 0.3154. Figure 8c shows the cumulative probability of the zoom lens versus the MTF at a magnification of M = −0.1150. The minimum value of the design plus MTF tolerance is 0.3838. Figure 8d show the cumulative probability versus the MTF curve of the zoom lens at a magnification of M = −0.1317. The minimum value of the design plus MTF tolerance is 0.3234. Figure 8e shows the curve for the cumulative probability versus MTF at a magnification of M = −0.1482. The minimum value of the design plus MTF tolerance is 0.3948. Figure 8f shows the curve of the cumulative probability versus MTF of the zoom lens at a magnification of M = −0.1690. The minimum value of the design plus MTF tolerance is 0.3428.

Based on the above analysis results, when the probability of successful manufacturing is above 2σ (97.7%), the image quality is within the acceptable range, and the goal of 30% can be achieved. Finally, the analysis is carried out to determine whether the tolerance range used is within the acceptable range. We compare the results to the used and acceptable tolerance ranges. Table 5 shows a comparison of the design plus MTF tolerance at different magnifications. The analysis results were all within the acceptable tolerance range.

### 4.3. Zoom Lens Resolution Analysis

Table 6 shows a comparison of the lowest resolution for different zoom positions. The lowest resolution of Zoom 1 at the wide-angle end is located at 0.82 tangential field of view (FOV). Considering that the image sensor can resolve the image at MTF > 0.3. In the design plus tolerance at the central FOV and 0.82 tangential FOV, the spatial frequency at MTF ≅ 0.3 is taken as the calculation. If the tolerance is added to the central FOV design, the MTF is 0.3009 and the spatial frequency is 257 lp/mm, then the minimum resolvable width of the image space is 1.946 µm. Furthermore, it multiplied by the system’s inverse magnification of 11.834, the minimum resolvable width of the object space is 23.029 µm. If 0.82 tangential FOV is designed with a tolerance, the MTF is 0.3013 and the spatial frequency is 157 lp/mm, then the minimum resolvable width of the image side is 3.185 µm. Multiplying this value by the system’s inverse magnification of 11.834, a minimum resolvable width of the object side is 37.691 µm. Since the effective photographic area is 1.840 mm × 1.040 mm, and multiplied by the inverse magnification of the system of 11.834, the photographic area of the object space is 21.775 mm × 12.308 mm. Based on the same analysis approach, the minimum resolution of other Zoom positions is described as follows:

(a) The lowest resolution for Zoom 2 position is located at a 1.0 tangential FOV. When the MTF of the central field of view design plus the tolerance is 0.3015, its spatial frequency is 210 lp/mm, so the minimum resolvable width of the image side is 2.381 µm, and multiplied by the system inverse magnification of 10.163, the minimum resolvable width of the object side can be obtained as 24.198 µm. When the 1.0 tangential field of view is designed with tolerance, MTF is 0.3019 and its spatial frequency is 152 lp/mm, the image side resolution width is 3.289 µm, and the resolution width of the object space is 33.426 μm, and the photographic area of the object is 18.700 mm × 10.570 mm.

(b) The lowest resolution of Zoom 3 position is located at a 1.0 tangential FOV. When the MTF of the central field of view design plus the tolerance is 0.3003 and the spatial frequency is 215 lp/mm, the minimum resolvable width of the image side is 2.326 µm. The inverse magnification of the above system is 8.696, and the minimum resolvable width of the obtained object is 20.227 µm. If 1.0 tangential field of view is designed with tolerance, MTF is 0.3009 and the spatial frequency is 163 lp/mm, then the resolution width of the image space is 3.067 µm, the resolution width of the object space is 26.671 µm, and the photographic area of the object space is 16.001 mm × 9.044 mm.

(c) The lowest resolution of Zoom 4 position is located at a 1.0 tangential FOV. If the MTF of the central FOV with a tolerance is 0.3023, and the spatial frequency is 205 lp/mm, then the minimum resolvable width of the image side is 2.439 µm. The inverse magnification of the above system is 7.593, and the minimum resolvable width of the object is 18.519 µm. When the 1.0 tangential FOV is designed with a tolerance, MTF is 0.3003 and the spatial frequency is 140 lp/mm, the resolution width of the image side is 3.571 µm, the resolution width of the object side is 27.115 µm, and the photographic area of the object side is 13.971 mm × 7.897 mm.

(d) The lowest resolution of Zoom 5 position is located at a 1.0 radial FOV. If the central field of view is designed with a tolerance, MTF is 0.3010 and the spatial frequency is 212 lp/mm, then the minimum resolvable width of the image side is 2.358 µm. Multiplying the inverse magnification of the above system by 6.748 gives a minimum resolvable width of the object of 15.912 µm. When the 1.0 radial FOV is designed with a tolerance, MTF is 0.3001 and the spatial frequency is 154 lp/mm, the resolution width of the image side is 3.247 µm, the resolution width of the object side is 21.911 µm, and the photographic area of the object side is 12.416 mm × 7.018 mm.

(e) The lowest resolution for Zoom 6 position is located at 0.82 tangential FOV. If the central field of view is designed with a tolerance, MTF is 0.3028 and the spatial frequency is 194 lp/mm, then the minimum resolvable width of the image side is 2.577 µm. The inverse magnification of the above system is 5.917, and the minimum resolvable width of the object side is 15.248 µm. If the 1.0 radial FOV is designed with a tolerance, MTF is 0.3001 and the spatial frequency is 154 lp/mm, the resolution width of the image side is 3.650 µm, the resolution width of the object side is 21.597 µm, and the photographic area of the object side is 10.887 mm × 6.154 mm.

## 5. Conclusions

Although international manufacturers, such as Olympus and Fujifilm etc., have many patents related to capsule endoscopy [16,17,18,19,20,21]. However, they lack the production of zoom lens endoscopes. In this work, we present an optical design of a 2× zoom lens used for a capsule endoscope. To reduce the production cost and weight, the number of zoom lens elements is less than five. A smaller F-number means a larger aperture and more incoming light, so there will be more stray light, but also a smaller depth of field. The F-number range used in this work is from F/2.8 to F/3.5. Since the capsule endoscope is mainly used to observe the abnormal condition of the intestinal wall, its object surface is not an ideal plane, which is close to the curved surface. Therefore, this study uses the object surface as the curved surface to design, which will be closer to the real situation. The design results show that the maximum optical distortion at the position of zoom 1 is −14.89%. Comparing from zoom 1 to zoom 6, the minimum value of relative illumination is 71.8% at Zoom 1 position. At the wide-angle end, a full viewing angle is 143° and a photographic area is 21.775 mm × 12.308 mm at Zoom 1 position, and a minimum photographic resolution is 23–38 µm from the center to the edge. At Zoom 6 position, the full viewing angle is 60°, the photographic area is 10.887 mm × 6.154 mm, and the minimum photographic resolution is 15–22 µm from the center to the edge. In this work, an image sensor with 1-million pixels and a pixel size of 1.4 µm is selected to design a zoom lens and apply it to the capsule endoscope, which can achieve the same imaging quality as that of the traditional endoscope. We propose a new 2× zoom capsule endoscope design to reduce manufacturing cost and achieve minimally invasive surgery in a resource limited environment.

## Figures and Tables

**Table 1:** Lens design data for 2× zoom lenses.

Surface No.	Surface Type	Radius (mm)	Thickness (mm)	Material	Full Aperture (mm)
Object	Sphere	15	10		
1	Sphere	−39.8878	0.8000	NLAK34_SCHOTT	6.0000
2	Sphere	2.2953	1.4238		3.8045
3	Asphere	1.6832	1.2000	PEIO	3.4216
4	Asphere	1.5473	d_4_		2.4192
Stop	Sphere	Infinity	0.1000		1.0647
6	Asphere	1.9349	1.3360	POLEFINH	1.2329
7	Asphere	−6.0669	d_7_		1.5288
8	Asphere	2.6530	1.4431	POLEFINH	2.3434
9	Asphere	67.8418	d_9_		2.3469
10	Sphere	Infinity	0.8000	NBK7_SCHOTT	2.2278
11	Sphere	Infinity	0.1000		2.1147
Image	Sphere	Infinity	0.000		2.1109

**Table 2:** Comparison of maximum optical distortion at different magnifications.

Different Magnifications and Field of Views (FOV)	Maximum Optical Distortion
M = −0.0845 (FOV = 143°)	−14.89%
M = −0.0984 (FOV = 114°)	−8.50%
M = −0.1150 (FOV = 93°)	−4.64%
M = −0.1317 (FOV = 79°)	−2.49%
M = −0.1482 (FOV = 69°)	−1.24%
M = −0.1690 (FOV = 60°)	1.45%

**Table 3:** Comparison of horizontal and vertical video distortion at different magnifications.

	Video Distortion	
Zoom Positions	Horizontal Video Distortion	Vertical Video Distortion
Zoom 1 (FOV = 143°)	3.31%	8.19%
Zoom 2 (FOV = 114°)	1.98%	4.98%
Zoom 3 (FOV = 93°)	1.30%	3.08%
Zoom 4 (FOV = 79°)	0.98%	2.07%
Zoom 5 (FOV = 69°)	0.83%	1.53%
Zoom 6 (FOV = 60°)	0.696%	0.996%

**Table 4:** Comparison of lateral chromatic aberrations at different magnification positions.

Different Zoom Positions and FOVs	Maximum Value of Lateral Color (Red Curve)	Maximum Value of Lateral Color (Green Curve)
M = −0.0845 (FOV = 143°)	0.001506 mm	−0.00081 mm
M = −0.0984 (FOV = 114°)	−0.001396 mm	0.000839 mm
M = −0.1150 (FOV = 93°)	−0.001397 mm	0.000627 mm
M = −0.1317 (FOV = 79°)	−0.001368 mm	0.000518 mm
M = −0.1482 (FOV = 69°)	−0.001311 mm	0.000440 mm
M = −0.1690 (FOV = 60°)	−0.001400 mm	0.000302 mm

**Table 5:** Comparison of the design plus MTF tolerance at different magnifications.

Different Zoom Positions	Minimum Value of the Design Plus MTF Tolerance
M = −0.0845	0.3176
M = −0.0984	0.3154
M = −0.1150	0.3838
M = −0.1317	0.3234
M = −0.1482	0.3948
M = −0.1690	0.3428

**Table 6:** Comparison of the lowest resolution for different zoom positions.

Zoom Positions	MTF Value	Spatial Frequency	Resolution Width of the Image Side	Photographic Area (mm^2^)
Zoom 1	0.3013	157 lp/mm	3.185 µm	21.775 × 12.308
Zoom 2	0.3019	152 lp/mm	3.289 µm	18.700 × 10.570
Zoom 3	0.3009	163 lp/mm	3.067 µm	16.001 × 9.044
Zoom 4	0.3003	140 lp/mm	3.571 µm	13.971 × 7.897
Zoom 5	0.3001	154 lp/mm	3.247 µm	12.416 × 7.018
Zoom 6	0.3001	154 lp/mm	3.650 µm	10.887 × 6.154

## Data Availability

Not applicable.
